# Dabrafenib: A New Therapy for Use in BRAF-Mutated Metastatic Melanoma

**Published:** 2014-05-01

**Authors:** Suzanne McGettigan

**Affiliations:** Abramson Cancer Center, University of Pennsylvania, Philadelphia, Pennsylvania

Melanoma, the most dangerous form of skin cancer, accounts for the majority of skin cancer–associated mortality. The American Cancer Society (ACS) has estimated that there will be 76,100 new cases of melanoma diagnosed in 2014, with an estimated 9,710 deaths from melanoma (ACS, 2014). The incidence of melanoma has been rising over the past 3 decades. Melanoma is the most common malignancy diagnosed in Caucasian women aged 20 through 29 and second only to breast cancer in women aged 30 through 34 (Howlader et al., 2013). Risk factors associated with the development of melanoma include skin type, hair and eye color, family and personal history of skin cancers, and most notably, UV exposure from both natural and artificial sources.

When diagnosed at an early stage, the 5-year survival rate associated with melanoma is greater than 90%; however, individuals diagnosed with stage IV melanoma have a 1-year survival rate of only 25% and a dismal 5-year survival rate of 10%.

The clinical management of advanced melanoma has changed dramatically in recent years. Prior to 2010, the only US Food and Drug Administration (FDA)-approved therapies for stage IV melanoma were high-dose interleukin-2 and dacarbazine, both of which were approved based on phase II clinical trial data. In 2011, two therapies were approved by the FDA for use in unresectable stage III and IV melanoma, based on improvements in overall survival noted in phase III trials. Ipilimumab (Yervoy) is a monoclonal antibody targeting CTLA-4, augmenting T-cell production and replication. Vemurafenib (Zelboraf) is a targeted therapy indicated in the treatment of *BRAF* V600E-mutated melanoma.

Dabrafenib (Tafinlar) is one of two agents that were approved by the FDA in March 2013, adding another weapon into the still limited armamentarium against metastatic melanoma. Dabrafenib is indicated for use in *BRAF* V600E-mutated metastatic melanoma (GlaxoSmithKline, 2014). Although it has a similar indication and a similar target as vemurafenib, the side-effect profile and the dosing of dabrafenib are different. It is critical that oncology advanced practitioners (APs) are aware of these differences and able to present this important information to their patients.

## Mechanism of Action

*BRAF* is an oncogene. Mutated forms of *BRAF*, including V600E, result in a constitutively activated *BRAF* kinase that may stimulate tumor growth. *BRAF* mutations occur in approximately 50% to 60% of melanomas; approximately 95% of melanomas that harbor a *BRAF* mutation are characterized as having a *BRAF* V600E mutation (Hocker & Tsao, 2007). Other *BRAF* mutations do occur, but at much lower rates. Dabrafenib is a kinase inhibitor that inhibits *BRAF* V600E mutation–positive melanoma. It is important to stress that dabrafenib should not be used in patients with *BRAF* wild-type melanoma, as in vitro studies have demonstrated a proliferative effect in nonmutated *BRAF* melanomas following exposure to *BRAF* inhibitors.

## Dosing

The recommended dose of dabrafenib is 150 mg orally twice daily (Table 1); medication should be taken on an empty stomach (either 1 hour before a meal or 2 hours after). Dabrafenib is metabolized by CYP2C8 and CYP3A4. Dabrafenib induces CYP3A4 and thus may alter the metabolism of other medications metabolized through this pathway by reducing the bioavailability of substrates. Of particular note in patients with advanced melanoma are dexamethasone, warfarin, and hormonal contraceptives. Additionally, medications that decrease gastric pH in the upper GI tract, such as proton pump inhibitors, may decrease dabrafenib bioavailability. Advanced practitioners should evaluate patient medication lists prior to initiating therapy with dabrafenib and initiate substitutions of medications when appropriate.

**Table 1:**
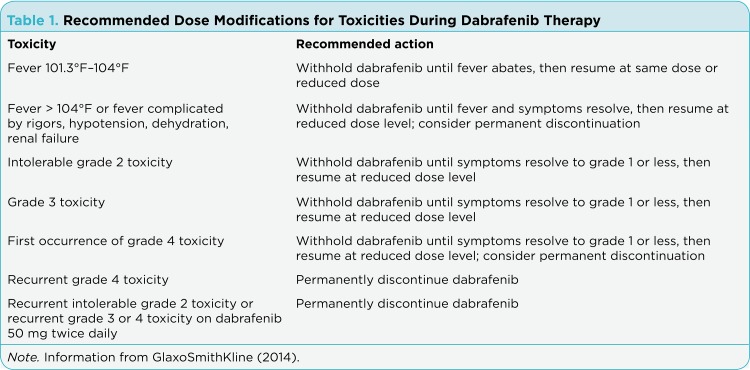
Table 1. Recommended Dose Modifications for Toxicities During dabrafenib Therapy

## Clinical Trials

In a phase I dose escalation trial enrolling 184 patients with solid tumors possessing a *BRAF* V600E or V600K mutation, a dose of 150 mg twice daily was recommended (Falchook et al., 2012). At this dose level, an overall response rate was seen in 69% of *BRAF* V600E-mutated melanoma (36 patients) and 78% of *BRAF* V600K-mutated melanoma (27 patients), confirmed in 50% and 56%, respectively (Falchook et al., 2012). Median relapse-free survival of 5.5 months was seen in patients with a *BRAF* mutation. Although no maximum tolerated dose was identified, a dose of 150 mg twice daily was selected for the phase II trials because near-maximum pharmacodynamic effect was present, and increased toxicity was noted in doses above 200 mg twice daily.

BREAK-2 was a multicenter single-arm phase II trial that enrolled 92 patients with centrally confirmed *BRAF* V600E- or V600K-mutated metastatic melanoma (Ascierto et al., 2013). Patients with brain metastases were excluded from this trial, whereas prior therapy was allowed. A dose of 150 mg twice daily was utilized. Confirmed response was reported in 45 (59%) patients in the V600E-mutated group; confirmed partial response was reported in 2 (13%) patients in the V600K-mutated group (investigator assessed). Stable disease was reported in 16% and 44% of patients, respectively. The median progression-free survival (PFS) was 6.3 and 4.5 months in the V600E- and V600K-mutated groups, respectively; median overall survival was 13.1 and 12.9 months (Ascierto et al., 2013).

BREAK-MB was a multicenter open-label phase II trial that enrolled patients with *BRAF* V600E- or V600K-mutated metastatic melanoma and brain metastases (Long et al., 2012). A total of 172 patients were randomized to two groups: Cohort A included patients without prior brain-directed therapy, while cohort B included those with disease progression following brain-directed therapy. Both groups received dabrafenib 150 mg twice daily until disease progression, unacceptable toxicity, or death. Overall intracranial response rates in V600E-mutated patients were 39.2% and 30.8% in cohorts A and B, respectively, with an additional stable disease rate of 42% and 58%; overall intracranial response rate in V600K-mutated patients was 6.7% and 22.2% in cohorts A and B, respectively, with an additional stable disease rate of 27% and 28% (Long et al., 2012).

Lastly, BREAK-3 was a large multicenter open-label randomized phase III clinical trial that enrolled patients with previously untreated (other than with interleukin-2) *BRAF* V600E-mutated stage IV melanoma (Hauschild et al., 2012). A total of 250 patients were randomized to receive dabrafenib 150 mg twice daily or dacarbazine 1,000 mg/m^2^ every 21 days; crossover was allowed for patients randomized to the dacarbazine arm. Patients with valvular abnormalities or abnormal left ventricular ejection fraction were excluded. Initial analysis occurred when the primary study objective was met. Median PFS was 5.1 months for dabrafenib and 2.7 for dacarbazine; the ORR was 50% and 7%, respectively, with stable disease seen in an additional 42% and 48%, correlating with a 39% improvement in OS in favor of dabrafenib (Hauschild et al., 2012). An updated analysis, 6 months later, demonstrated a PFS of 6.9 months for dabrafenib and 2.7 months for dacarbazine; the PFS was 4.3 months for patients who crossed over to dabrafenib following dacarbazine (Hauschild et al., 2013).

## Adverse Event Management

The most common side effects noted in patients receiving dabrafenib include hyperkeratosis, headache, pyrexia, arthralgia, papilloma, alopecia, and palmar-plantar erythrodysesthesia (PPE, also known as hand- foot syndrome). The most serious adverse events noted in patients receiving dabrafenib include squamous cell carcinomas of the skin, tumor promotion in *BRAF* wild-type melanoma, serious febrile reactions, hyperglycemia, and uveitis/iritis. Other rare but serious toxicities reported include hypophosphatemia, increased alkaline phosphatase, hyponatremia, pancreatitis, and interstitial nephritis (GlaxoSmithKline, 2014).

The most common cutaneous developments in the setting of dabrafenib therapy include cutaneous squamous cell carcinoma; keratocanthoma; melanoma; and other keratotic cutaneous lesions, including verrucal lesions, Grover’s disease, and palmar-plantar hyperkeratosis (Anforth et al., 2012; Belum et al., 2013). Photosensitivity and alopecia can also occur. Patients should undergo dermatologic screening prior to initiation of dabrafenib therapy, every 2 months during therapy, and every 6 months thereafter. The AP should counsel patients about the importance of self-examination for the detection of new skin lesions, sun-protective behaviors, and management strategies for PPE. It is not necessary to withhold the dose or to dose-reduce dabrafenib following the development of cutaneous skin lesions; in the dose-escalating study, all squamous cell carcinomas were well-differentiated without recurrence or metastatic disease (Anforth et al., 2012). Twenty percent of patients developed any grade PPE; dose reductions may be necessary for intolerable grade 2 or 3 PPE (GlaxoSmithKline, 2014).

The AP should advise patients to report fevers immediately, as 28% of patients develop pyrexia during dabrafenib therapy. Patients should be counseled about the symptoms of serious febrile reactions, including hypotension, rigors, chills, dehydration, and renal failure in the absence of an identifiable infectious etiology. Common symptoms that accompany fevers include rash and arthralgias (Lee et al., 2012). Throughout the clinical development of dabrafenib, the median onset of fever was 11 days, with a median duration of 3 days (GlaxoSmithKline, 2014). Dabrafenib should be withheld for fevers of 101.3°F to 104°F; when fever is resolved, dabrafenib can be resumed at the same dose or a reduced dose (Table 1). For fevers > 104°F or those accompanied by rigors, hypotension, dehydration, or renal failure, dabrafenib may be withheld until resolved and then resumed with a dose reduction (Table 2). The AP should ensure that the patient is staying well-hydrated to prevent complications of serious febrile reactions such as hypotension and renal dysfunction.

**Table 2:**
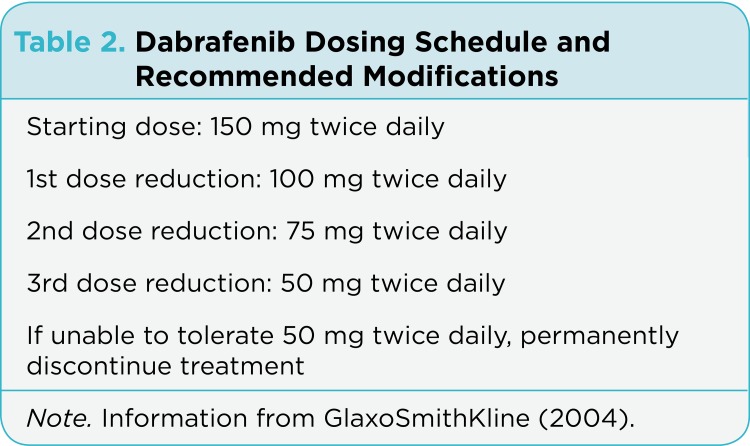
Table 2. Dabrafenib Dosing Schedule and Recommended Modifications

A single-institution report of febrile episodes in patients receiving dabrafenib indicated that neither dose reductions nor antipyretic therapies were successful; corticosteroid treatment was the only effective treatment in managing fevers and febrile reactions associated with dabrafenib (Lee et al., 2012). In the absence of suspected infectious etiology of fever, corticosteroids should be initiated in patients experiencing fevers or febrile syndrome.

The AP should monitor serum glucose levels closely in patients with preexisting diabetes or hyperglycemia; serious hyperglycemia occurred in 6% of patients. Patients should be educated about the symptoms associated with hyperglycemia. Patients with preexisting diabetes or hyperglycemia may require increases in antidiabetic medications (GlaxoSmithKline, 2014).

Patients must be monitored for symptoms of uveitis or iritis throughout dabrafenib therapy, including changes in vision, photophobia, or eye pain; in clinical trials, treatment included referral to ophthalmology and initiation of steroid and mydriatic drops (GlaxoSmithKline, 2014). In patients with a glucose-6- phosphate dehydrogenase (G6PD) deficiency, monitor closely for the development of hemolytic anemia.

Dabrafenib is categorized as a pregnancy category D agent; in animal data, fetal toxicity was noted. Effects on spermatogenesis have been observed in animal models. Patient should be advised to stop nursing during therapy with dabrafenib. Advise patients to avoid pregnancy during dabrafenib therapy and for 4 weeks following dabrafenib. As dabrafenib can render hormonal therapies ineffective, patients should be counseled to utilize nonhormonal methods of contraception (GlaxoSmithKline, 2014).

## Future Directions

Ongoing clinical trials investigating the use of dabrafenib in combination with other targeted therapies are ongoing. The combination of dabrafenib and trametinib (Mekinist) was approved by the FDA in January 2014. Additionally, the use of dabrafenib in non–*BRAF* V600E-mutated melanoma continues to be investigated. Trials on the use of dabrafenib in other solid tumors including *BRAF* V600E/K-mutated colorectal cancer, *BRAF* V600E-mutated non–small cell lung cancer, and *BRAF* V600E-mutated thyroid cancer are also ongoing.

## Summary

Dabrafenib is an oral therapy approved for use in *BRAF* V600E-mutated metastatic melanoma. *BRAF* inhibition has been demonstrated to be an effective therapeutic target in melanoma; dabrafenib joins vemurafenib, an oral *BRAF* inhibitor, which was FDA approved in 2011 (Tawbi & Kirkwood, 2012).

Dabrafenib extends progression-free and overall survival in patients with unresectable stage III and IV melanoma. Despite these results, the effect is temporary, and continued improvements in therapeutic options for these patients remain necessary.

The AP plays an important role in the initiation and monitoring of patients receiving oral therapies, including dabrafenib. While manageable, the side effects of this therapy can be frightening to patients, their caregivers, and members of the health-care team. The AP is in a unique position to educate patients and their caregivers about the toxicities of this therapy, to make dose adjustments as needed, and to initiate appropriate supportive care.
